# En Route to Targeted Ribosome Editing to Replenish Skin Anchor Protein LAMB3 in Junctional Epidermolysis Bullosa

**DOI:** 10.1016/j.xjidi.2023.100240

**Published:** 2023-11-10

**Authors:** Bjoern Wimmer, Andreas Friedrich, Katharina Poeltner, Genevieve Edobor, Claudia Mosshammer, Gazmend Temaj, Adriana Rathner, Thomas Karl, Jan Krauss, Joerg von Hagen, Christopher Gerner, Michael Breitenbach, Helmut Hintner, Johann W. Bauer, Hannelore Breitenbach-Koller

**Affiliations:** 1 Department of Biosciences and Medical Biology, University of Salzburg, Salzburg, Austria; 2 Faculty of Pharmacy, College UBT, Prishtina, Kosovo; 3 Institute of Biochemistry, Johannes Kepler University of Linz, Linz, Austria; 4 SKM-IP PartGmbB, Munich, Germany; 5 Merck KGaA, Gernsheim, Germany; 6 ryon-Greentech Accelerator, Gernsheim, Germany; 7 Department of Analytical Chemistry, Faculty of Chemistry, University of Vienna, Vienna, Austria; 8 Joint Metabolome Facility, University of Vienna, Vienna, Austria; 9 Department of Dermatology and Allergology, University Hospital Salzburg, Salzburg, Austria

**Keywords:** Genodermatoses, Drug development, Blistering disease, Epidermolysis bullosa, Genetic diseases

## Abstract

Severe junctional epidermolysis bullosa is a rare genetic, postpartum lethal skin disease, predominantly caused by nonsense/premature termination codon (PTC) sequence variants in *LAMB3* gene. *LAMB3* encodes LAMB3, the β subunit of epidermal–dermal skin anchor laminin 332. Most translational reads of a PTC mRNA deliver truncated, nonfunctional proteins, whereas an endogenous PTC readthrough mechanism produces full-length protein at minimal and insufficient levels. Conventional translational readthrough-inducing drugs amplify endogenous PTC readthrough; however, translational readthrough-inducing drugs are either proteotoxic or nonselective. Ribosome editing is a more selective and less toxic strategy. This technique identified ribosomal protein L35/uL29 (ie, RpL35) and RpL35-ligands repurposable drugs artesunate and atazanavir as molecular tools to increase production levels of full-length LAMB3. To evaluate ligand activity in living cells, we monitored artesunate and atazanavir treatment by dual luciferase reporter assays. Production levels of full-length LAMB3 increased up to 200% upon artesunate treatment, up to 150% upon atazanavir treatment, and up to 170% upon combinatorial treatment of RpL35 ligands at reduced drug dosage, with an unrelated PTC reporter being nonresponsive. Proof of bioactivity of RpL35 ligands in selective increase of full-length LAMB3 provides the basis for an alternative, targeted therapeutic route to replenish LAMB3 in severe junctional epidermolysis bullosa.

## Graphical abstract

**Figure:**
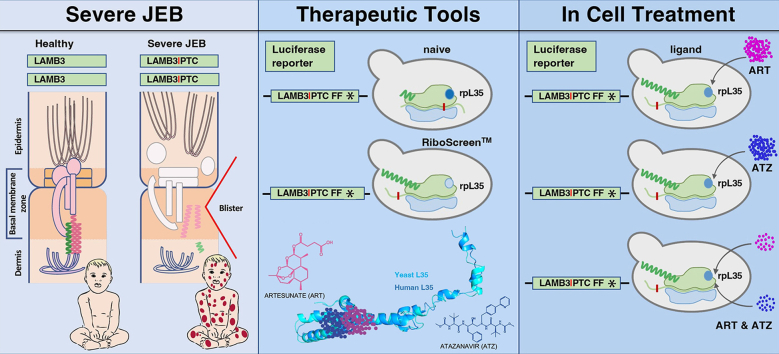
Graphical abstract shows therapeutic avenue to customize replenishment of skin anchor protein LAMB3 in severe Junctional Epidermolysis bullosa.

## Introduction

Epidermolysis bullosa (EB) comprises a heterogeneous group of rare genetic dermatoses, and subtypes of EB result from different inherited loss-of-function sequence variants in genes encoding structural proteins of the skin (Bardhan et al, 2020). Junctional EB (JEB) is caused by the absence or functional loss of heterotrimeric laminin 332 (Lm332), type XVII collagen, or integrin α6β4 (Bardhan et al, 2020; Has et al, 2020), all components of or associated with hemidesmosomes. Hemidesmosomes and associated proteins reside at the apical site of basal keratinocytes (KCs) and form proteinous skin anchors, which provide flexible but resilient molecular rivets for adherence of the epidermis to the dermis.

**Figure 1:**
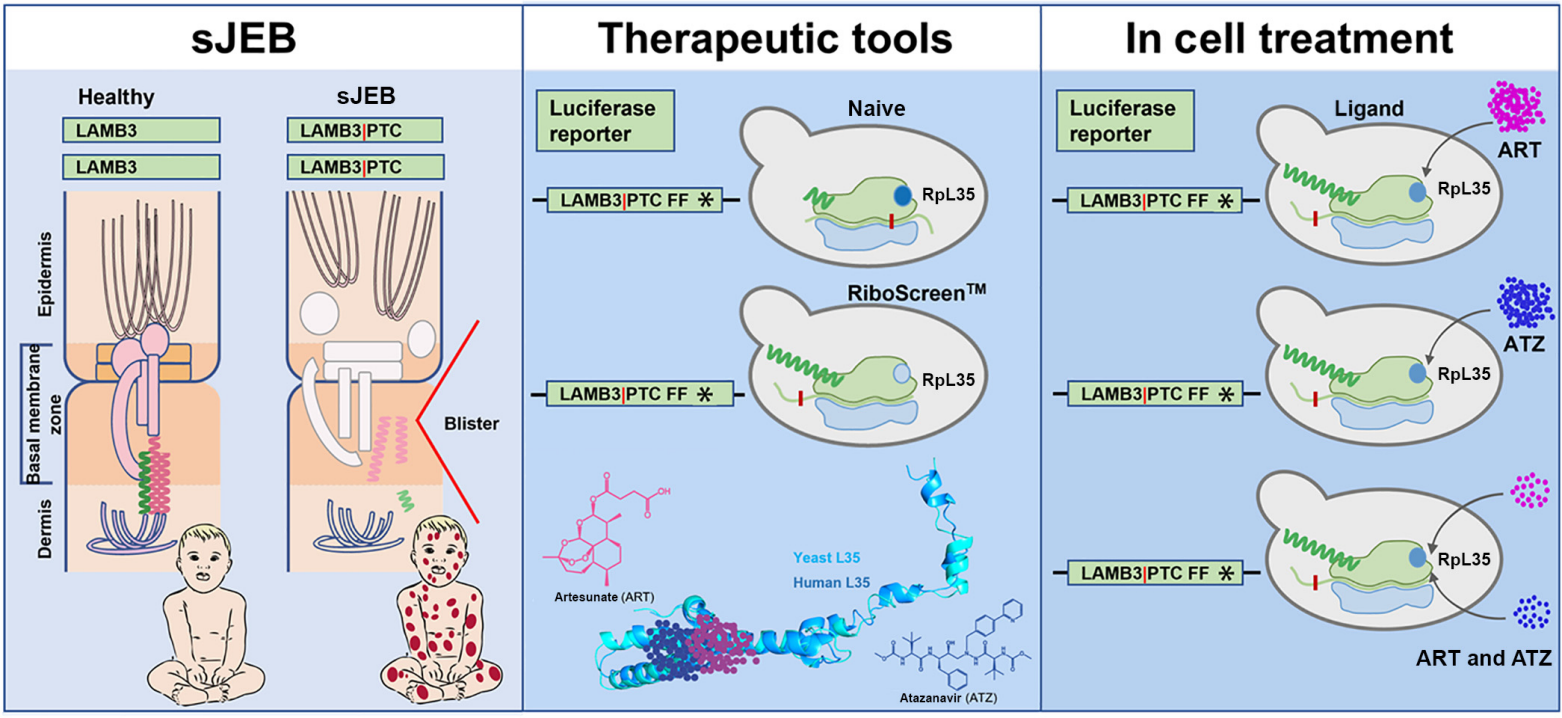
**Therapeutic avenue to customize replenishment of skin anchor protein LAMβ3 in sJEB.** The left side of the first panel shows healthy status. In the skin, the epidermis and dermis are linked by a flexible but resilient multiprotein skin anchor (pink). At the apical side of basal keratinocytes, skin anchor proteins condense to form hemidesmosome (boxed orange), a molecular rivet that provides attachment of the epidermis to the dermis. On a structural level, this is achieved by trimeric laminin 332 (Lamb3 in green and Lama3 and Lamg2 in bright pink) within the extracellular basement membrane zone, which links an appendage of HD to the collagen network of the underlying dermis. The right side of the first panel depicts the pathologic status. Homozygous PTC sequence variants in LAMB3 (ie, LAMB3-PTC; red mark) lead to loss of full-length LAMB3 protein and failure to assemble trimeric Laminin 332 skin anchor (muted green, muted pink). Loss of laminin 332 triggers disintegration and degradation of skin anchor proteins (white). Consequently, the epidermal–dermal junction is disrupted (red arrowhead), and large blisters form on the skin and internal epithelia, leading to the early demise of patients. Currently, no approved therapy is available. The second panel shows therapeutic tools for systemic ribosome editing as delivered by RiboScreen technology. First, RpL35 was identified as a target ribosomal protein for boosting production levels of full-length LAMB3. Repurposable drugs artesunate and atazanavir were identified as small-molecule ligands of RpL35. The third panel shows the cellular effect of treatment with RpL35 ligands artesunate and atazanavir. Treatment with either drug or combinatorial treatment of both drugs at reduced dosage increased the production levels of full-length LAMB3 but not of control reporter proteins (not shown). HD, hemidesmosome; PTC, premature termination codon; sJEB, severe junctional epidermolysis bullosa.

Laminins are multifunctional glycoproteins that control the homeostasis of cells and tissues by their structural, adhesive, signaling, and mechanical functions (Aumailley, 2013). Loss of Lm332 severely diminishes the hemidesmosomal skin anchors and leads to severe JEB (sJEB). Therefore, in sJEB upon minimal trauma, skin and mucosal layers split at the epidermal–dermal boundary, that is, the basement membrane zone; large blisters form; and the massive disruption of the protective barrier results in inflammation, impairment of wound healing, and systemic extracutaneous ailment (Bardhan et al, 2020; De Rosa et al, 2019; Has et al, 2020). Sequence variants in any one of 3 genes *LAMA3, LAMB3,* and *LAMC2*, encoding the laminin α3, β3, and γ2 subunit chains of Lm332 (LAMα3, LAMβ3, and LAMγ2), respectively, generate absence or pronounced depletion of functional Lm332. *LAMB3R635X* is the predominant mutant allele observed in sJEB, and patients homozygous for *LAMB3R635X* rarely survive the first 2 years after birth (Hammersen et al, 2016; Keith et al, 2020) (Figure 1).

When a single nucleotide change within a reading frame of an mRNA generates a premature termination codon (PTC) sequence variant, also called a nonsense codon sequence variant, this unscheduled stop codon signal triggers the binding of the translation termination complex, aborting of protein synthesis, release of a truncated protein from the ribosome, and degradation of PTC mRNA by the nonsense-mediated mRNA decay (NMD) pathway (Brown et al, 2015; Kurosaki et al, 2019; Lindeboom et al, 2016). However, during protein synthesis, fidelity of decoding of both cognate codons and nonsense codons cannot be achieved at an absolute level. Indeed, amino acid misincorporations during translation have been reported to occur at a frequency that is about tenfold higher (0.01–1%) at nonsense codons than at natural stop codons (0.001–0.1%) (Drummond and Wilke, 2009). Hence, at low frequency, a PTC is misread not as a stop codon but as a sense codon to result in innate, basal endogenous PTC suppression. On a molecular level, this is achieved by base pairing of a near-cognate aminoacyl-transfer RNA with 2 of the 3 nucleotides of an unscheduled stop codon to outcompete termination complex binding at the PTC. Consequently, translation elongation continues in the correct ribosomal reading frame, producing a full-length protein (Lombardi et al, 2020).

Accordingly, for *LAMB3R635X* mRNA, scant endogenous levels of PTC readthrough are reported, with full-length protein only detectable with signal amplifying assays. Notably, this does not provide those minimal levels of full-length LAMβ3 (LAMβ3FL) to form adequate levels of Lm332s and hemidesmosomes, respectively, necessary to ensure basal levels of functional epidermal–dermal adhesion (Gache et al, 2001).

Great effort has been made to explore the PTC fidelity drawback as a therapeutic approach to boost production levels of full-length protein, which resulted in development of readthrough-inducing drugs (RTIDs) (reviewed in Dabrowski et al [2021, 2018]). Classical RTIDs are exemplified by aminoglycoside (AG) antibiotics, which target the decoding center of bacterial ribosome and have been used for decades to alter the accuracy of prokaryotic pathogen protein translation, thus promoting the production of nonfunctional pathogen proteins (Prokhorova et al, 2017). However, reduced affinity of AGs to eukaryotic ribosomes fostered their use as RTIDs in therapeutic intervention in PTC-associated diseases (Bukowy-Bieryllo et al, 2016; Keeling et al, 2014; Prokhorova et al, 2017), often in combination with NMD-suppressing drugs, which protect PTC mRNAs from decay (McHugh et al, 2020; Yu et al, 2022). In a parallel fashion, macrolide antibiotics (MALs), which target the peptidyl transferase center of the ribosome to promote PTC readthrough, have been explored as RTIDs (Caspi et al, 2016; Dabrowski et al, 2018). However, both AGs and MALs fail to discriminate between disease-associated PTCs, natural stop codons, and endogenous regulatory PTCs (Dabrowski et al, 2018; Wangen and Green, 2020), and prolonged use of these RTIDs is toxic (Prayle and Smyth, 2010). In addition, alternative development of non-AG RTIDs, exemplified by ataluren, often did not result in superior performance and, depending on the experimental approach used, delivered conflicting results as to their specificity for PTC readthrough compared with cognate stop codon readthrough (Bolze et al, 2017; Wang et al, 2022). This leaves the most severe form of EB, sJEB, without approved therapy, although on the level of cellular assays, recent studies have shown that augmenting endogenous readthrough with even small amounts of additional LAMβ3FL can reduce the severity of the molecular phenotype, including restoration of the Lm332 complex and hemidesmosomal function (Keith et al, 2020; Kwong et al, 2020; Lincoln et al, 2018). These latter findings are substantiated by reports on a first series of pioneering clinical trials, which examined gentamicin topical treatment in patients with JED with *LAMB3PTC* sequence variants (Kwong et al, 2020), or by single cycle intravenous administration and/or intramuscular injection (Hammersen et al, 2019). Both these therapeutic avenues showed increased expression of LAMβ3, restoration of the Lm332 complex at the dermal–epidermal junction, and improved wound healing. However, a more long-term favorable outcome upon treatment was not reported for patients homozygous or compound heterozygous for *LAMB3PTC* sequence variants. Recently, Mosallaei et al (2022) reported on a clinical trial (NCT03526159 and NCT04140786) that monitored the level of molecular analysis, immunohistochemistry, and wound healing in one course of gentamicin at low-dose intravenous treatment (7.5 mg/kg for 14 days) or high-dose intravenous treatment (10 mg/kg for 24 days) in 5 pediatric patients with JEB (age range from 3 months to 10 years, 80% females). Patients were heterozygous for at least 1 confirmed PTC variant in either *LAMα3* or *LAMB3*, including 1 patient being compound heterozygous for 2 different PTC sequence variants in *LAMB3* (referred to as patient CHZPTC in the remaining parts of this paper) (Mosallaei et al, 2022). This systemic therapeutic regime resulted in all patients demonstrating an increased readthrough of PTC variants to generate full-length proteins, restored functional Lm332 at the epidermal–dermal junction, and promoted wound closure during the 3-month study period. All patients, including patient CHZPTC, completed the study, and no ototoxic or nephrotoxic effects or antilaminin antibodies were detected within the course of the study. Importantly, in skin samples of patient CHZPTC, obtained 1 month after 14 days of low-dose intravenous treatment, LAMβ3FL was present at 45% of the control level (Mosallaei et al, 2022), whereas previous cell culture studies testing high doses of gentamicin for translational readthrough-inducing drug (TRID) activity for 24 or 36 hours reported considerable less readthrough activity, between 2 and 10% of control, reviewed in Dabrowski et al (2018) and Keith et al (2020). In conclusion, Mosallaei et al (2022) suggest that a single cycle of intravenous gentamicin may be a safe and readily available therapy in the short term for such a population of patients with JEB. However, AGs, including gentamicin, are ribosomal ligands that globally affect translation (Wangen and Green, 2020), and it will prove challenging to establish long-term safety and efficacy studies required to obtain approval for continuous systemic therapy for these general TRIDs.

A targeted and alternative approach to adjusting protein production levels to therapeutic needs and at physiologically meaningful levels is presented by RiboScreen technology, which targets ribosomal protein function to tailor the efficiency of mRNA translation of a disease-associated protein (Bauer et al, 2013). RiboScreen, in a cost-efficient first step, screened a library of variant yeast strains, each edited by genetic depletion of one or the other of the 80 eukaryotic ribosomal proteins, for boosting production levels of a LAMβ3FL reporter protein. RiboScreen identified 1 variant strain, with ribosomes functionally demarcated by reduced gene dosage of ribosomal protein L35/uL29 (referred to as RpL35 in the remaining parts of this paper) and which promoted increased production levels of LAMβ3FL reporter protein (Bauer et al, 2013). Then, RpL35 was employed as a target ribosomal protein to identify in a second step small molecules binding to RpL35. Two approved drugs, artesunate and atazanavir, were identified as RpL35 ligands, and both molecules bind to RpL35 in solution (Rathner et al, 2021).

In this study, in a next step of RiboScreen technology, we report that treatment of naïve yeast vehicles with either RpL35 ligand, artesunate or atazanavir, or their combinatorial treatment at reduced drug dosage selectively amplified production levels of LAMβ3FL reporter protein up to 200%.

## Results

### Treatment with RpL35 ligands artesunate and atazanavir promote increased production levels of LAMβ3FL

The dual luciferase reporter assay tool developed for the original screening assay (Bauer et al, 2013) in this study was recycled to test the effect of treatment with RpL35 ligands artesunate and atazanavir in naïve yeast vehicles, that is, to boost the production levels of LAMβ3FL encoded by a parent *LAMB3R635X* mRNA.

Therefore, for every individual dual luciferase experiment, wild-type (WT) strain BY4743 was cotransformed with the Renilla (gene *REN)* luciferase reporter plasmid pLM 161, encoding the internal control protein reporter Renilla and 1 or other of 4 Firefly (gene *FF*) luciferase–based reporter plasmids, pLM 168, which carries C-terminally *FF-*tagged *LAMB3* sequence; pLM 169, which carries C-terminally *FF-*tagged *LAMB3R635X* sequence; pLM 162, which carries the *FF* luciferase sequence; and pLM167, which carries the *FFY53X* sequence (Bauer et al, 2013). This generated coexpressed reporter protein pairs in yeast vehicles, with REN expression levels serving as normalizer. C-terminal FF (Firefly protein) tagging ensured that the protein expression level of full-length protein only is reported, both for the native LAMB3 and FF proteins as well as for their respective full-length variants LAMβ3FL and FFFL (full-length FF protein), encoded by the respective PTC mRNAs.

**Figure 2:**
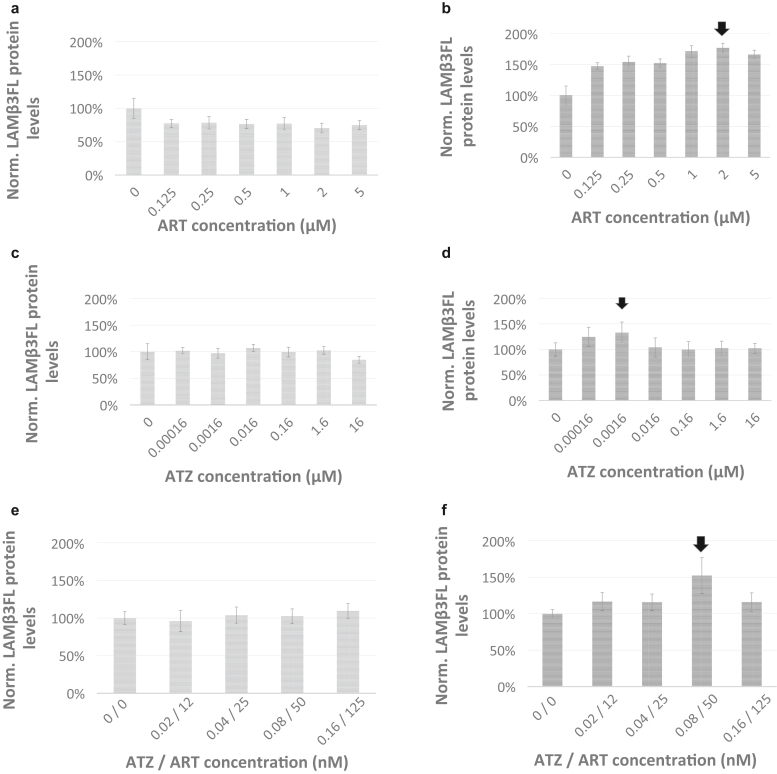
**Treatment with RpL35 ligands ART and ATZ triggers increased production levels of LAMβ3FL in yeast cellular assays.** Treatments of yeast vehicles transformed with dual luciferase reporter plasmids expressing C-terminally FF-tagged LAMB3 (Lamβ3 protein reporter) and REN (Renilla protein reporter) are shown in **a**, **c**, and **e**. Treatments of yeast vehicles transformed with dual luciferase reporter plasmids expressing C-terminally FF-tagged mutant LAMB3 protein (R635X), which reports the production of LAMB3FL reporter protein, and REN (Renilla protein reporter) are shown in panel **b**, **d**, and **f**. Vehicles were grown for 18 h under treatment with ART and ATZ or combined treatment of ATZ and ART. Reporter activity was measured in RLUs, and reporter protein expression levels were calculated as the FF signal to REN signal ratio and normalized to the mean FF signal to REN signal ratio set to 100% in untreated cells. Mean normalized reporter protein expression levels based on luciferase activity are shown with error bars, presenting the data as mean ± 1 SD. Varying concentrations as indicated on the x-axis were tested for ART, ATZ, and ATZ/ART. Three replicates per well were collected to assess RLUs for a given drug treatment condition, and at least 4 wells were measured per experiment (n = 12). The black arrow indicates treatment conditions delivering maximal response. ART, artesunate; ATZ, atazanavir; h, hour; LAMβ3FL, full-length LAMβ3; RLU, Relative Light Unit.

Inspection of Figure 2a and Table 1 indicates that normalized WT LAMβ3 protein expression levels on the basis of luciferase-integrated signal intensities show a minor but significant decrease to 0.8-fold in protein production level upon 0.125, 0.25, 0.5, 1, 2, and 5 μM artesunate treatment.

**Table 1:** Summary Statistics of Reporter Protein Expression Levels upon Treatment with ART, ATZ, ART/ATZ, and ERY

Treatment	Concentration (μM)	Normalized LAMB3 Levels (%)	*P*-Value	Normalized LAMB3FL Levels (%)	*P*-Value	Normalized FF Levels (%)	*P*-Value	Normalized FFFL Levels (%)	*P*-Value
ART	0	100%		100%		100%		100%	
ART	0.125	77%	3.5 × 10^−4^	147%	9.8 × 10^−6^	94%	9.7 × 10^−2^	104%	5.6 × 10^−1^
ART	0.25	78%	7.4 × 10^−4^	154%	4.0 × 10^−9^	93%	4.1 × 10^−2^	88%	4.2 × 10^−2^
ART	0,5	77%	2.8 × 10^−4^	152%	4.6 × 10^−10^	93%	2.4 × 10^−2^	100%	9.4 × 10^−1^
ART	1	77%	4.2 × 10^−4^	171%	1.5 × 10^−11^	93%	4.5 × 10^−2^	95%	6.7 × 10^−1^
ART	2	71%	2.5 × 10^−5^	**176%**	**1.1 × 10**^**−12**^	90%	6.1 × 10^−3^	94%	1.1 × 10^−1^
ART	5	75%	1.4 × 10^−4^	166%	5.1 × 10^−12^	86%	1.3 × 10^−3^	87%	3.5 × 10^−2^
ATZ	0	100%		100%		100%		100%	
ATZ	0.00016	102%	7.6 × 10^−1^	125%	2.8 × 10^−3^	107%	3.5 × 10^−1^	102%	6.0 × 10^−1^
ATZ	0.0016	97%	7.1 × 10^−1^	**133%**	**6.0 × 10**^**−4**^	102%	7.8 × 10^−1^	99%	8.8 × 10^−1^
ATZ	0.016	107%	3.1 × 10^−1^	104%	5.9 × 10^−1^	101%	8.9 × 10^−1^	109%	4.7 × 10^−2^
ATZ	0.16	99%	9.1 × 10^−1^	100%	9.8 × 10^−1^	101%	9.3 × 10^−1^	107%	4.1 × 10^−2^
ATZ	1.6	103%	7.1 × 10^−1^	103%	6.3 × 10^−1^	109%	2.1 × 10^−1^	106%	3.2 × 10^−1^
ATZ	16	85%	2.9 × 10^−2^	102%	6.8 × 10^−1^	109%	2.3 × 10^−1^	94%	2.5 × 10^−1^
ART and ATZ	0/0	100%		100%		100%		100%	
ART/ATZ	0.02/12	96%	4.4 × 10^−1^	117%	1.7 × 10^−3^	93%	6.3 × 10^−2^	101%	8.4 × 10^−1^
ART/ATZ	0.04/25	104%	4.1 × 10^−1^	116%	2.2 × 10^−3^	91%	1.4 × 10^−2^	105%	2.5 × 10^−1^
ART/ATZ	0.08/50	103%	5.2 × 10^−1^	**152%**	**7.2 × 10**^**−4**^	88%	1.3 × 10^−3^	106%	3.3 × 10^−1^
ART/ATZ	0.16/125	109%	2.8 × 10^−2^	116%	5.0 × 10^−3^	88%	4.8 × 10^−4^	106%	2.5 × 10^−1^
ERY	0	100%		100%		100%		100%	
ERY	0.0001	99%	8.6 × 10^−1^	112%	1.6 × 10^−4^	102%	3.2 × 10^−1^	113%	1.7 × 10^−1^
ERY	0.001	102%	5.4 × 10^−1^	122%	3.6 × 10^−6^	95%	7.2 × 10^−2^	123%	2.7 × 10^−2^
ERY	0.01	104%	3.1 × 10^−1^	134%	1.9 × 10^−9^	93%	5.8 × 10^−3^	123%	3.6 × 10^−2^
ERY	0.1	99%	7.2 × 10^−1^	141%	3.4 × 10^−7^	92%	2.0 × 10^−2^	112%	3.8 × 10^−1^
ERY	1	95%	2.4 × 10^−1^	146%	4.3 × 10^−13^	94%	1.0 × 10^−2^	**200%**	**4.9 × 10**^**−6**^
ERY	2	87%	8.4 × 10^−4^	**166%**	**8.5 × 10**^**−8**^	94%	1.1 × 10^−2^	157%	2.4 × 10^−5^
ERY	4	90%	1.8 × 10^−2^	151%	5.7 × 10^−9^	91%	1.1 × 10^−3^	156%	2.2 × 10^−4^

Abbreviations: ART, artesunat; ATZ, atanazavir; ERY, erythromycin; LAMB3FL, full-length LAMB3.

Boldfaced data indicate maximal responses to drug treatments of LAMB3FL and FFFL. To assess the statistical significance of change in reporter protein expression upon small-molecule treatment, a Student´s *t*-test was applied to the normalized reporter protein expression levels. 0 indicates untreated conditions, and respective treatment conditions are listed. A *P* ≤ .01 was considered significant.

However, in comparison with basal levels of LAMβ3FL expressed in untreated vehicles resulting from endogenous readthrough, treatment with artesunate delivered a dose-dependent increase of LAMβ3FL signal, which peaked significantly at 1.8-fold increase at 2 μM artesunate (Figure 2b and Table 1). We note that artesunate at optimal treatment conditions delivers a twofold increase in the production level of LAMβ3FL, which is identical to the fold increase reported in the original Riboscreen upon depletion of RpL35 (Bauer et al, 2013). We conclude that treatment with RpL35 ligand artesunate mirrors the effect of reduced functional availability of RpL35 in boosting the protein production level of LAMβ3FL (Bauer et al, 2013).

Treatment with atazanavir did not change expression levels of WT LAMβ3 signals under any study conditions (Figure 2c and Table 1). However, there was a dose-dependent increase in the production level of LAMβ3FL signal, which peaked significantly at 1.3-fold at 0.0016 μM atazanavir treatment (Figure 2d and Table 1).

Comparing artesunate with atazanavir treatment, we note several distinct features. For both artesunate and atazanavir treatment, the dose–response curves indicate that the respective treatment conditions generating the maximal response reported in this study are close to the optimal concentrations possible. Second, upon artesunate treatment, increased 1.8-fold production levels of LAMβ3FL signal are accompanied by a small decrease in WT LAMβ3 signals at all treatment concentrations used. Third, atazanavir boosts production levels of LAMβ3FL by 1.3-fold, with no significant change in protein reporter expression signals for the WT LAMβ3 variant. This suggests an orthogonal mode of molecular action of these 2 RpL35 ligands, supported by our finding that these 2 small molecules have overlapping but not identical binding sites on the RpL35 protein (Rathner et al, 2021).

### Artesunate and atazanavir act synergistically to boost the production levels of LAMβ3FL

We reasoned that the combinatorial drug treatment of artesunate and atazanavir might at reduced drug dosage also boost the production levels of LAMβ3FL. Therefore, we treated yeast vehicles at combinatorial concentrations of 0.02, 0.04, 0.08, and 0.16 nM atazanavir and 12, 25, 50, and 125 nM artesunate, respectively. Inspection of Figure 2e and Table 1 shows that at all combined treatments, WT LAMβ3 signals do not change. In contrast, there is a dose-dependent increase in protein production levels of LAMβ3FL (Figure 2f and Table 1), peaking at 1.5-fold at combinatorial treatment of 0.08 nM atazanavir and 50 nM artesunate. We conclude that to arrive at a similar fold increase in the production level of LAMβ3FL as provided by either drug alone, combinatorial treatment allows a reduction of small-molecule dosage for both artesunate and atazanavir by 2 orders of magnitude.

### Artesunate and atazanavir do not boost protein production levels of FFFL

Next, we queried protein production levels of FF and FFFL to investigate whether treatment of RpL35 ligands artesunate and atazanavir or their combinatorial treatment also boosts the production level of an unrelated protein derived from a PTC parent mRNA.

**Figure 3:**
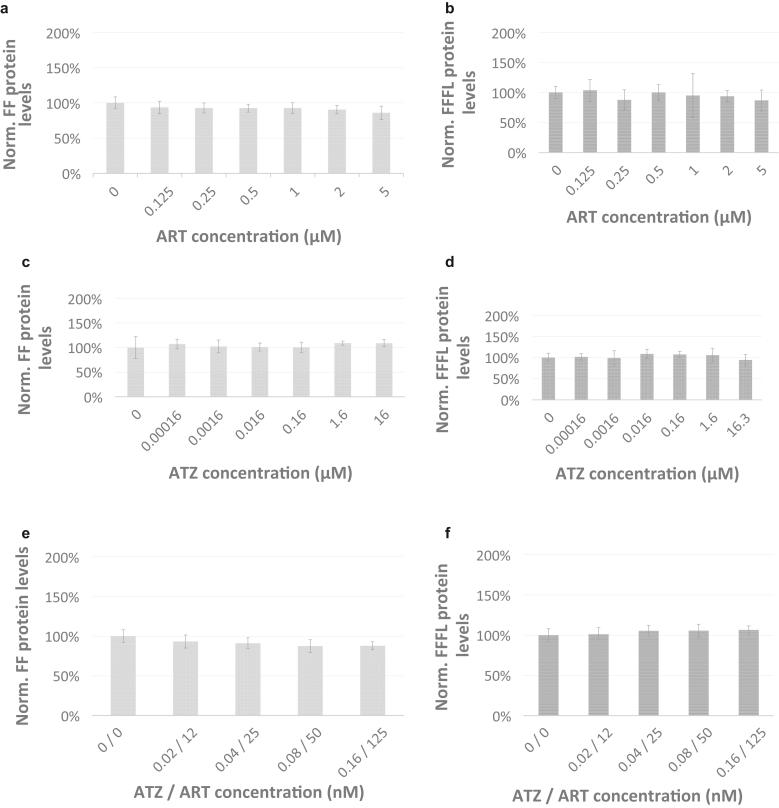
**Treatment with RpL35 ligands ART and ATZ does not trigger increased production levels of FFFL in yeast cellular assays.** Treatment of yeast vehicles transformed with luciferase reporter plasmids expressing Firefly (FF protein reporter) and Renilla (REN protein reporter) are shown in **a**, **c**, and **e**. Treatments of yeast vehicles transformed with dual luciferase reporter plasmids expressing mutant Firefly protein (Y53X), which reports production of full-length FF reporter protein (ie, FFFL) and REN (Renilla protein reporter) are shown in **b**, **d**, and **f**. Vehicles were grown for 18 h under treatment with ART and ATZ or combined treatment of ATZ and ART. Reporter activity was measured in RLUs, and reporter protein expression levels were calculated as the FF signal to REN signal ratio and normalized to the mean FF signal to REN signal ratio in untreated cells. Mean normalized reporter protein expression levels based on luciferase activity are shown with error bars, presenting the data as mean ± 1 SD. Varying concentrations as indicated on the x-axis were tested for ART, ATZ, and ATZ/ART. Three replicates per well were collected to assess RLUs for each drug treatment condition, and at least 4 wells were measured per experiment (n = 12). The black arrow indicates the treatment condition delivering maximal response. ART, artesunate; ATZ, atazanavir; h, hour; RLU, Relative Light Unit.

Inspection of Figure 3 and Table 1 shows that WT FF protein expression levels are neither altered by treatment with artesunate (Figure 3a) nor with atazanavir (Figure 3c). In addition, combinatorial treatment with artesunate and atazanavir did not alter protein expression levels of the FF reporter protein (Figure 3e). We conclude that treatment with RpL35 small-molecule ligands artesunate and atazanavir at the conditions used in this study does not alter FF protein expression levels.

Inspection of Figure 3b and d shows that both RpL35 ligands (artesunate and atazanavir, respectively) and at all concentrations used do not promote a significant increase in protein production levels of FFFL (Table 1). This is similar to what has been found for a depletion of RpL35, which neither alters native FF protein expression levels nor FFFL protein levels (Bauer et al, 2013). Thus, RpL35 modification—generated either by genetic depletion of RpL35 (Bauer et al, 2013) or treatment with RpL35 ligands (Rathner et al, 2021)—favorably modifies the recoding of a PTC located within a *LAMB3*mRNA architecture but not within that of a *FF* mRNA species.

### Erythromycin, a general PTC readthrough drug, boosts the production levels of both LAMβ3FL and FFFL proteins

RpL35 resides on the large ribosomal subunit and with a long protrusion extends into the ribosomal RNA folds forming the protein exit tunnel, through which the nascent polypeptide chain migrates to emerge from the ribosome (Rathner et al, 2021). MALs, such as erythromycin, bind to a ribosomal RNA pocket in the ribosomal exit tunnel of bacterial ribosomes and act as a general translation elongation inhibitor of pathogens in the human host (Svetlov et al, 2021; Wilson, 2014). Analogous to AGs, the MAL erythromycin has also been used as a therapeutic TRID, in particular for treatment of familial adenomatous polyposis to repair PTC sequence variants in the *APC* (adenomatous polyposis coli) gene (Kariv et al, 2020).

**Figure 4:**
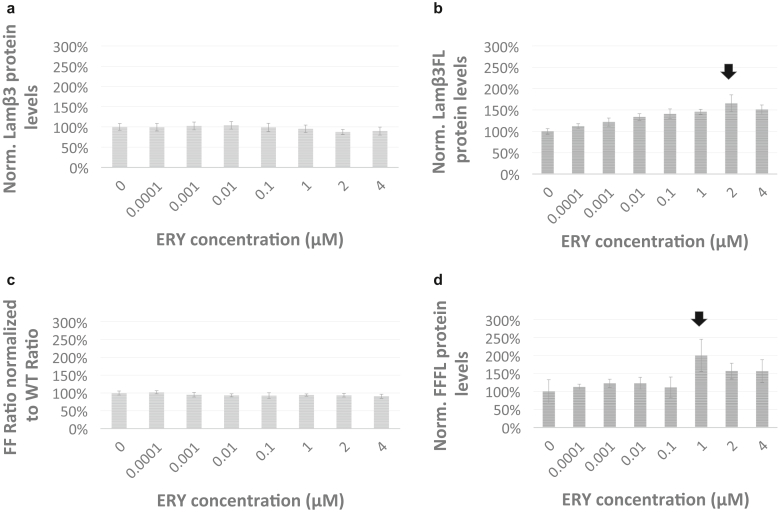
**Treatment with macrolide antibiotic ERY nonselectively triggers increased production levels of LAMβ3FL and FFFL in cellular assays.** (**a**) Treatment of yeast vehicles transformed with dual luciferase reporter plasmids expressing C-terminally FF-tagged LAMB3 (LAMB3 protein reporter) and REN (Renilla protein reporter). (**b**) Treatment of yeast vehicles transformed with dual luciferase reporter plasmids expressing C-terminally FF-tagged mutant LAMB3 protein (R635X), which reports the production of LAMB3FL reporter protein (LAMB3FL) and REN (Renilla protein reporter). (**c**) Treatment of yeast vehicles transformed with luciferase reporter plasmids expressing Firefly (FF protein reporter) and Renilla (REN protein reporter). (**d**) Treatment of yeast vehicles transformed with dual luciferase reporter plasmids expressing mutant Firefly protein (Y53X), which reports production of full-length FF reporter protein (FFFL) and REN (Renilla protein reporter). Vehicles were grown for 18 h under treatment with ERY. Reporter activity was measured in RLUs, and reporter protein expression levels were calculated as the FF signal to REN signal ratio and normalized to the mean FF signal to REN signal ratio in untreated cells. Mean normalized reporter protein expression levels based on luciferase activity are shown with error bars, presenting the data as mean ± 1 SD. Varying concentrations as indicated on the x-axis were tested for ERY. Three replicates per well were collected to assess RLUs for a given drug treatment condition, and at least 4 wells were measured per experiment (n = 12). The black arrow indicates the treatment condition delivering maximal response. ERY, erythromycin; h, hour; LAMβ3FL, full-length LAMβ3; RLU, Relative Light Unit.

In this study, we wished to investigate whether protein expression levels of LAMβ3 and FF reporter species as well as LAMβ3FF and FFFL, respectively, are responsive to treatment with erythromycin, reported to act as a nondiscriminatory PTC readthrough drug (Caspi et al, 2016). We observed that at no treatment conditions used did erythromycin change the production levels of WT LAMβ3 or WT FF (Figure 4a and c and Table 1). However, erythromycin showed dose-dependent stimulation of production levels of LAMβ3FL, which peaked at 1.7-fold at 2 μM erythromycin treatment (Figure 4b and Table 1). This is similar to what was obtained with artesunate treatment (Figure 2b). Also observed was a dose-dependent stimulation of production levels of FFFL, which peaked twofold at 1 μM erythromycin treatment (Figure 4d). Our results confirm the findings of other studies, which have shown a nonselective mode of action for erythromycin in increasing the production levels of full-length protein from different PTC parent mRNAs (Chen et al, 2015; Seefeldt et al, 2021; Thompson et al, 2004; Vázquez-Laslop and Mankin, 2018).

## Discussion

So far, several in vivo and ex vivo molecular therapy approaches aiming to augment compromised skin protein function or to restore physiological levels of functional proteins levels in EB, including sJEB, have been tested (reviewed in the study by Welponer et al [2021]), although none has been approved and licensed for clinical use in EB. In this study, in cellular protein expression assays, we investigated ribosomal protein RpL35 ligands, repurposable drugs artesunate and atazanavir, for their potential to increase production levels of full-length skin anchor protein LAMβ3FL. Drug repurposing has been suggested as a possible strategy for swift development of medicines for rare diseases because safety profiling is available from clinical and regulatory assessments, as are pharmacokinetics, dosage, quality control, and production process (Kort and Jovinge, 2021; Tambuyzer et al, 2020). This reduces overall risk, costs, and timeline for clinical approval of drugs for rare diseases without treatment options (Pushpakom et al, 2019; van den Berg et al, 2021).

On the flip side, repurposable drugs, although having obtained approval from the authorities for one disease, could adversely affect patients with another disease, presenting with a pathophysiology unrelated to the original indication. Ideally, to circumvent possible pitfalls in drug repurposing, strategies have to be designed to decrypt drug action regarding pathway engagement and cellular mechanism of action. For example, Zhao et al (2021) obtained such a molecular activity forecast by cataloging the effects of 704 clinically approved drugs for their impact on cellular NMD, which degrades transcripts with nonsense/premature stop codons. The authors showed that most of the cataloged Food and Drug Administration–approved drugs caused insignificant effects on NMD, and small sets of drugs were found to have a mild effect on NMD, either promoting NMD inhibition or NMD potentiation. NMD inhibition can also enhance the effectiveness of TRID nonsense suppression therapy, which allows a translational readthrough of the PTC to produce full-length functional proteins. Repurposable drug artesunate mapped into the class of compounds inhibiting NMD but only with a mild effect on NMD inhibition of some but not all endogenous NMD substrates tested (Zhao et al, 2021). Therefore, studying artesunate as a ribosomal protein ligand and as a potential selective TRID in essence should report on its activity to modulate ribosomal function to promote increased production levels of LAMβ3FL from a *LAMB3PTC* substrate.

In this study, we have shown that treatment with either drug, artesunate at low micrometer concentration and atazanavir at low nanometer concentration, promotes an increase of up to 200% in the production level of LAMβ3FL but not that of unrelated FFFL. Moreover, there is a synergistic mode of action of these compounds because combined treatment at a reduced dosage of both molecules also promoted increased production levels of LAMβ3FL, similar to what was observed for single-molecule treatment at respective higher dosages and again with no effect on boosting FFFL protein. Selectivity of artesunate and atazanavir is further supported by our observation that treatment with a general readthrough drug, the MAL antibiotic erythromycin, promotes increased protein expression levels of both LAMβ3FL and FFFL reporter proteins. This suggests a selective and targeted mode of action of RpL35 ligands artesunate and atazanavir in bypassing the PTC signal during translation of the *LAMB3PTC* mRNA but not that of the *FF PTC* mRNA.

To prevent termination of translation at a PTC, a near cognate transfer RNA (tRNA) must be accepted at the ribosomal decoding site (A-site), a process termed PTC readthrough (Floquet et al, 2012; Wangen and Green, 2020). On a molecular level, the best characterized TRIDs are antibiotics of the AG and MAL class (Böttger et al, 2001; Poehlsgaard et al, 2005; Prokhorova et al, 2017) and ataluren, chemically a substituted oxadiazole, which is structurally neither related to AGs nor to MALs (Borgatti et al, 2020; Dabrowski et al, 2021).

Binding of AGs at the ribosomal A-site distorts the geometry of the decoding site; decreases elongation rates; lowers fidelity of codon–anticodon recognition; and promotes misreading of sense codons, PTC codons, and cognate stop codons by incorporation of near cognate tRNAs (Garreau de Loubresse et al, 2014; Prokhorova et al, 2017; Ying et al, 2019). In the case of a PTC codon, this allows bypass of the PTC codon with translation to continue and to produce a full-length protein from a PTC mRNA. MALs, exemplified by erythromycin, plug the ribosomal protein exit tunnel. There they modulate the passage of the growing elongating peptide chain and promote allosteric effects, which alter the functional properties of the catalytic center of the ribosome, thereby decreasing the fidelity of decoding. Consequently, this too favors PTC misreading by a near cognate tRNA but also misreading of cognate stop codons and sense codons (Caspi et al, 2016; Seefeldt et al, 2021; Vázquez-Laslop and Mankin, 2018).

Ataluren promotes misreading of PTCs (Roy et al, 2016) by a different mechanism because kinetic studies revealed that ataluren acts as a competitive inhibitor of the release factor complex, thereby preventing preferentially termination of translation at a PTC, with more minor effects on cognate stop codon readthrough (Huang et al, 2022; Ng et al, 2021). Treatment with ataluren would then favor the incorporation of a near cognate tRNA on a kinetic level (Roy et al, 2016) rather than providing altered allosteric control of amino acid–tRNA loading during the elongation phase of translation, as delivered by AGs or MALs (Polikanov et al, 2018; Svetlov et al, 2021; Wangen and Green, 2020). Whereas both AGs and MALs efficacy is sensitive to the sequence context of the PTC (Bukowy-Bieryllo et al, 2016; Pranke et al, 2018; Vázquez-Laslop and Mankin, 2018), ataluren shows less sensitivity to sequence context (Michorowska, 2021; Tutone et al, 2019). Indeed, complementary mode of action of AGs and ataluren was demonstrated by their orthogonal activity on PTC readthrough (Ng et al, 2021). However, all these RTIDs fail to discriminate between disease-conferring and endogenous regulatory PTC signals as well as cognate stop codons (Dabrowski et al, 2018; Tutone et al, 2019; Wangen and Green, 2020). With no other therapeutic intervention available, AGs, MALs, and ataluren are tested as TRIDs for treating hereditary PTC diseases, exemplified by the clinical application of ataluren for PTC sequence variants in Duchenne muscular dystrophy (McDonald et al, 2022) and for compassionate use in JEB (Orlowski et al, 2023).

A first appreciation of a more targeted molecular mode of action to recode a PTC sequence variant by altered ribosomal function may be derived from proteomic studies querying the effects of altered functional availability of ribosomal protein S14/uS11. This showed that altered elongation rates changed the production levels of selected proteins, all characterized by specific mRNA architectures, conferred by transcript length, codon bias, and distinct sequence elements (Boussaid et al, 2021). Recently, elongation rates were measured for the first time in living mammals (Gerashchenko et al, 2021) and were found to vary between 4 amino acids per second to 8 amino acids per second, depending on the type of tissue and metabolic state. This provides an estimate for a possible and physiologically relevant several-fold increase or decrease in protein production that could be achieved by ribosomes specialized to alter the elongation rates of selected mRNAs. We hypothesize that binding of artesunate to RpL35 might specifically decrease the ribosomal elongation rate for the parent *LAMB3*mRNA as observed in our study (Figure 2b). This is reminiscent of what has been observed for promiscuous downregulation of elongation rates upon AG treatment (Aguirre Rivera et al, 2021; Wohlgemuth et al, 2021). Such a scenario in the case of artesunate would customize increased recoding of a PTC within a *LAMB3* mRNA, thereby increasing the production level of LAMβ3FL. On the other hand, atazanavir may induce a complimentary RpL35-specific change of ribosome function, possibly related to release factor activity, as was observed for ataluren, however with *LAMB3*mRNA-specific PTC recognition. If so, an mRNA of unrelated architecture, for example, encoding FF or FF PTC, would be unresponsive to the RpL35-mediated specialization of ribosomes, and this is what we observed in this study.

The basal endogenous level of LAMβ3FL produced in patient cells with homozygosity for *LAMB3R635X* has been estimated to be 0.5% of WT level, and a targeted twofold increase in production level as reported in this study achieves the critical threshold of 1% WT level to provide an ameliorated EB-associated disease phenotype, as has been reported in several studies (Hammersen et al, 2016; Keith et al, 2020; Lincoln et al, 2018).

For clinical translation of RpL35 ligands artesunate and atazanavir (Rathner et al, 2021) as repurposable drugs, it is prudent to catalog their pathway engagement in known treatment regimes. Artesunate is a semisynthetic derivative of the natural compound artemisinin. In the active chemical moiety of artemisinin-type compounds, an endoperoxide bridge is cleaved in the presence of ferrous iron by a Fenton-type reaction to generate ROS, for example, superoxide anions, hydroxyl radicals, and other radical molecules (Berdelle et al, 2011). Ferrous iron is present in high amounts in erythrocytes where the malaria pathogen *Plasmodium falciparum* home in. The oxidative burst generated by artesunate in the erythrocyte setting overwhelms the phalanx of antioxidative enzymes expressed by the pathogen and so contributes to the bacteriostatic effect (Vasquez et al, 2021). Thus, the potency and rapid onset of action with few documented adverse effects make artesunate effective in eliminating the otherwise multidrug-resistant parasite. In comparison with that in WT cells, ferrous iron is typically present in excess in cancer cells (Brown et al, 2015). A heme-centric mechanism to explain artesunate activation and specificity in cancers was proposed by Wang et al (2018). These and other authors described that artesunate in cancer cells but not in normal cells blocks cancer cell signaling pathways whose enhanced activity is obligatory for driving malignant growth (reviewed in the study by Augustin et al [2020]). This is exemplified by studies that showed that artesunate treatment in cancer models mitigates the Wnt pathway (Geng et al, 2021), the phosphoinositide-3 kinase/protein kinase B/mTOR pathway (Xiao et al, 2020), as well as their downstream targets (Gong et al, 2022; Yin et al, 2020). In addition, artesunate was found to block angiogenesis (Wei and Liu, 2017), invasion (Farhan et al, 2021), and metastasis (Jin et al, 2022). In addition, artesunate attenuates inflammatory pathways by affecting TNF-α as well as TGF-β and NF-κB signaling pathways (Liu et al, 2021; Nunes et al, 2017). Moreover, artesunate has been described to promote cancer cell death by stimulating apoptosis and modulating autophagy (Chen et al, 2021). In contrast to this diverse research results using in vitro cancer cell models and animal studies, there are few reports on phase I antineoplasia trials investigating artemisinin-type drugs (Deeken et al, 2018; Trimble et al, 2020; von Hagens et al, 2019). Although artesunate was well-tolerated during treatment and toxicities were primarily low grade, the discrepancy between experimental and clinical data highlights the need for patient-centered research to clarify the impact of artemisinin derivatives in cancer treatment (Raffetin et al, 2018). The second RpL35 ligand studied reported in this paper, atazanavir, is an azapeptide HIV-1 protease inhibitor with activity against HIV-1 protease, a virus-specific enzyme catalyzing the proteolytic cleavage of the viral polyprotein precursors. The resulting individual functional proteins assemble into infectious HIV-1 particles. The mode of action of atazanavir is conferred by inhibitory binding to the HIV-1 protease active site, which prevents cleavage of the viral polyproteins and generates immature noninfectious viral particles (Fuster and Clotet, 2005). In comparison with other protease inhibitors, atazanavir shows good gastrointestinal tolerability and allows for once-daily dosing (De Clercq and Li, 2016). Information on pathway engagement of atazanavir is scarce, and studies in cell culture studies have shown that atazanavir may induce premature senescence (Basisty et al, 2020) and may unfavorably impact glucose metabolism in patients presenting with preconditioned insulin sensitivity (Kajogoo et al, 2021). However, the molecular mechanisms that mediate these effects have not been reported. Taken together, cataloging the known molecular engagements of RpL35 ligands artesunate and atazanavir suggests that these compounds may be developed as repurposed drugs for treatment of sJEB.

In addition, to further decrease risk and increase the success rate for clinical translation of artesunate and atazanavir as systemic drugs to treat sJEB, further considerations have to receive attention. First, known experimental routes to develop systemic therapy for treatment of sJEB may forecast steps of clinical translation of artesunate and atazanavir. In an elegant series of experiments, De Rosa et al (2019) contributed to the understanding of the pivotal question as to how in monogenic disease, repair of one mutant protein could trigger a multilayer response with the endpoint of wound healing in JEB, as observed by Mosallaei et al (2022), Kwong et al (2020), and Hammersen et al (2019). De Rosa et al (2019) and Fu et al (2022) started from the observation that for healthy skin function, the proteins establishing the epidermal dermal junction adhesive machinery have to sense adhesion-dependent mechanical cues, which are regulated by the Hippo signaling pathway. The core of the mechanical clue-sensing Hippo pathway is a kinase cascade that ultimately phosphorylates the major Hippo pathway downstream effectors, the transcription factors yes-associated protein (YAP), and TAZ (transcriptional coactivator with PDZ-binding motif). Phosphorylation inactivates the YAP/TAZ axis, thus inhibiting the transcription of their downstream target genes, which encode drivers of cell proliferation in stem cells and progenitor cells (Wang et al, 2018). De Rosa et al (2019) showed that active YAP is dramatically decreased in sJEB KCs and that in normal KCs, induced loss of LAMB3 compromised YAP activity and recapitulated the sJEB phenotype. It was then demonstrated that in sJEB primary KCs, *LAMB3* gene therapy rescues YAP activity and promotes epidermal stem cell supply in vitro and in vivo. It may be speculated that wound closure upon systemic one-course treatment with gentamicin as reported by Mosallaei et al (2022) was triggered by an increase in production levels of LAMβ3FL; structural restoration of the Lm332 complex; and consequently, functional restoration of the mechanosensing YAP/TAZ axis. Activity of the latter is a prerequisite for continuous stem cell supply in wound healing (Yang et al, 2020).

Second, comparative cataloging of altered mechanosensing pathway functions in *LAMB3* PTC model KCs and skin equivalents (of both); the effect of a homozygous PTC sequence variant in *LAMB3*; as well as treatment thereof with artesunate, atazanavir, and their combinatorial use may pave the way for refined safety profiling of these Food and Drug Administration–approved drugs. This strategy may serve a named person trial for long-term treatment of a patient otherwise left without curative therapy.

We conclude that combinatorial treatment of artesunate and atazanavir may now be developed to provide targeted and customized systemic therapy with improved drug efficacy and minimal side effects, in particular for long-term treatment of sJEB.

## Materials and Methods

### Chemicals

Components for media preparation for *E coli* and *Saccharomyces cerevisiae* were obtained from GIBCO-BRL (Paisley, Scotland) and Sigma-Aldrich (St. Louis, MO), respectively. Midi Plasmid DNA purification kit was bought from NucleoBond (Macherey-Nagl, Düren, Germany). Artesunate and atazanavir sulfate were purchased from Sigma-Aldrich, and erythromycin was purchased from Merck (Darmstadt, Germany). Stop&Glo Luciferase substrate and the recommended lysis buffer kit were purchased from Promega (Madison, WI).

### Strains and plasmids

Yeast strain BY4743 was obtained from the systematic genome-wide deletion collection (www.euroscarf.de). Yeast media, culture conditions, and the manipulation of yeast cells were as described (Rose et al, 1990). Yeast cells were either incubated in rich medium (YPD) or in synthetic minimal medium, with the appropriate nutrient supplements. For yeast transformation, lithium acetate protocol was followed (Ausubel, 1989).

Plasmids carrying luciferase reporter sequences have been described previously (Bauer et al, 2013). In short, parent vectors used for all experiments were YCplac33 harboring a *URA3* selection marker and YCplac111 harboring a *LEU2* selection marker (Gietz and Sugino, 1988). Firefly luciferase reporter gene (*FF)* (Promega), *FFY53X* as well as C-terminal FF-tagged *LAMB3*, and *LAMB3R635X* sequence were each cloned into YCplac33 under the control of ADH1 promotor and ADH1 terminator (Gray et al, 1982), generating plasmids pLM162, pLM167, pLM168, and pLM169, respectively (Bauer et al, 2013). Renilla (REN) luciferase control reporter sequence (Promega) was cloned into YCPlac111 vector, under the control of ADH1 promotor and ADH1 terminator, generating plasmid pLM164 (Bauer et al, 2013).

### Dual Luciferase recordings in a semiautomated 96-well assay

For every experimental dual luciferase setup, a diploid WT yeast strain (BY4743) was cotransformed at 1 μg/μl with the REN reporter plasmid pLM164 and 1 or the other of the 4 different FF reporter plasmids expressing *FF* sequence (pLM162), *FFY53X* sequence (pLM167), *LAMB3* sequence (pLM168), and *LAMB3R635X* (pLM169). This generated coexpressed protein reporter pairs in the WT strain. After cotransformation, a single freshly grown colony was used to start a precultivation culture by inoculating 3 ml of SC- Ura-Leu medium. After an incubation time of about 7 hours at 28 °C at 250 r.p.m. (Brunswick incubation shaker), these cultures were used to inoculate 25 ml of SC-Ura-Leu medium at optical density_600_ = 0.004. From one 25 ml culture, 4 replicates of 200 μl/well samples were transferred into 96-well plates, using transparent Greiner F 96-well plates, employing an Integra Assist Plus pipetting station. In the case of treatment, 40 μl of small-molecule solution of appropriate concentration was added to cover a range of nanometer to millimeter treatment, and in the case of control condition, 40 μl of medium was added. The 96-well plate was then covered with a lid; placed in a Styrofoam box equipped with a water reservoir to avoid evaporation; and then placed into an incubator for 18 hours at 28 °C at 250 r.p.m. Cell cultures were processed aiming at an optical density_600_ = 1.5.

A culture volume of 240 μl in a 96-well format allowed for harvesting 50 μl from each culture condition in triplicate, employing Integra Assist Plus pipetting station for transfer into a black and white 96-well plate (PerkinElmer) suitable for Luciferase measurements. The luciferase assays were performed with the Stop&Glo Dual-Luciferase Reporter Assay System, and the reagents were prepared as indicated by the supplier (Promega). For one dual luciferase readout per 50 μl sample, the FF reaction (50 μl of LARII) and the REN reaction (50 μl of Stop&Glo) were automatically and sequentially injected by the GloMax Luminometer, setting the volume of the GloMax injectors to 50 μl, with speed set to 200 μl/sec, duration to 2 seconds, integration to 10 seconds, readings to 1, and interval to 0.3 seconds; Firefly and Renilla luciferase readouts were recorded by the GloMax Luminometer and stored as CSV files, which were transferred into Excel files for statistical processing.

### Treatment conditions

Transformants were treated with small molecules for 18 hours, and treatment conditions were calculated such that in 240 μl well volume, a final concentration of small molecules dissolved in DMSO (≤0.02%) was achieved as follows: artesunate at 0.125, 0.25, 0.5, 1, 2, and 5 μM; atazanavir at 0.00016, 0.0016, 0.016, 0.16, 1.6, and 16 μM; combinatorial treatment of artesunate and atazanavir at concentrations of 12.5 and 0.02 nM, 25 and 0.04 nM, 50 and 0.08 nM, and 125 and 0.16 nM; and erythromycin at 0.1, 1, 10, and 100 nM and 1, 2, and 4 μM. For each treatment condition, 4 individual experiments were performed, with 3 technical replicates per individual experiment.

### Statistics

Raw data were exported as a CSV file from GlowMax luminometer, imported into an Excel file, and aggregated with secondary experimental data (date, volume, optical density_600_, treatment type, treatment condition). The respective FF and REN reporter protein expression signals were subjected to descriptive statistics (including Anderson–Darling normality test) employing Minitab, and then the data were tested for outliers using an implemented Dixon Q-testing. A *P* < .05 was considered statistically significant. The identified outliers were marked and excluded for further statistical analysis. To visualize a possible RpL35 ligand–induced fold change in luciferase reporter protein production levels upon treatment, we first calculated the mean ratio of the FF reporter signals to REN reporter signals for each individual treatment condition. Then, the ratios obtained for the individual treatment conditions were normalized to the respective mean ratio recorded for the untreated WT (control). This normalized dataset was now subjected to *t*-test analysis to evaluate the significance of a possible response to small-molecule treatment. These data were visualized and analyzed using Microsoft Excel, and data are presented as mean ± SD.

## Data availability statement

Data related to this article are provided in Supplementary Table S1.

## ORCIDs

Bjoern Wimmer: http://orcid.org/0000-0002-9969-1256

Andreas Friedrich: http://orcid.org/0000-0002-8272-2490

Katharina Poeltner: http://orcid.org/0000-0002-3379-5888

Genevieve Edobor: http://orcid.org/0000-0001-6330-7745

Adriana Rathner: http://orcid.org/0000-0003-2919-9407

Thomas Karl: http://orcid.org/0000-0001-8730-5330

Gazmend Temaj: http://orcid.org/0000-0003-4807-2938

Claudia Moßhammer: http://orcid.org/0000-0003-0555-5219

Helmut Hintner: http://orcid.org/0000-0001-9199-0991

Michael Breitenbach: http://orcid.org/0000-0003-0868-9036

Christopher Gerner: http://orcid.org/0000-0003-4964-0642

Jan Krauss: http://orcid.org/0000-0003-2040-5008

Joerg von Hagen: http://orcid.org/0000-0001-9810-3590

Johann W. Bauer: http://orcid.org/0000-0002-6085-9170

Hannelore Breitenbach-Koller: http://orcid.org/0000-0002-8387-6408

## Conflict of Interest

HB-K, JWB, HH, and JK are shareholders of KBHB Consult GmbH, which holds IP (EP 2 251 437 B1, CA2778355C, United States pending and DE102020120218A1, EP21783401.9, United States pending). The remaining authors state no conflict of interest.
